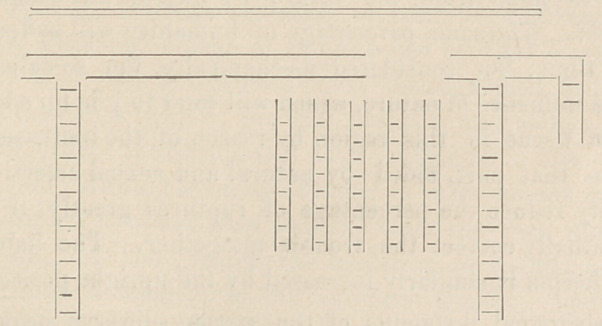# Disadvantages of the Upright Position

**Published:** 1884-03

**Authors:** S. V. Clevenger


### Selections.

---

DISADVANTAGES OF THE UPRIGHT POSITION. By S. V. CLEVENGER, M.D.

The immediate and remote causes of things have been and will be sought by thinkers who are not afraid to follow wherever facts lead them. The doctrine that there is no effect without an antecedent cause has met with fierce opposition from those who saw that the logical conclusions of correlated facts, such as are presented by Darwin, tended to the overthrow of puerile legends they believed in, and who were content to imagine that everything was causeless, or at least originated in some inscrutable way. The Arab, upon having the sidereal motions explained to him, said, "You trouble yourself greatly about things not intended for you to know. Even though what you tell me is true, the Koran leads us to believe otherwise. Mohammed taught us sufficient, and his followers can torture you out of your rationalism. Forbear your heretical facts!"

The mechanical nature of things inanimate, is as old in theory as Democritus, 500 B.C.; and Giordano Bruno, in A. D. 1600, for having amplified the Democritic idea, was burned at the stake. Kant granted a mechanical cosmogony, but in organic nature claimed *causæ finales*. The battle of *causæ efficientes* was fully won by Galileo, Copernicus, Kepler, Newton, Herschel, Laplace, etc., so far as the inanimate universe was concerned, but the mechanical conception of that which pertains to living things was hinted at by Aristotle. Geoffrey de St. Hilaire contended against Cuvier for the mutability of species and the monistic theory. Treviranus, Oken, Gœthe, Lamarck, and in our day, Darwin,

Hæckel, Huxley, have carried on the warfare. Herbert Spencer advanced a mechanical physiology and morphology. He has carried the conception into histology, and Cope into palæontology. The unity of the laws which control organic and inorganic nature are to-day fully recognized by those who stand in the front rank of investigators and thinkers, but not until completer text-books from the new standpoint shall have found their way into the hands of medical students and naturalists generally, will common recognition of the success of the mechanical idea be obtained.

Assuredly the teleological is a very lazy way of thinking. It amounts to taking things for granted as so, because they are so. It bars all inquiry, stops all investigation, and hands us bound hand and foot to ignorance and superstition.

Mechanical influences, such as impacts and strains, permanently altering animal organs, have been discussed by Professor E. D. Cope in the *American Naturalist*, in articles entitled "Origin of the Foot Structures of Ungulates," April, 1881; "Effects of Impacts and Strains on the Feet of Mammalia," July, 1881; by Alpheus Hyatt, "Transformations of Planorbis at Steinheim, with Remarks on the Effects of Gravity upon the Forms of Shells and Animals," June, 1882. In articles published in the January and February, 1881, numbers, I attempted a disquisition upon physical influences in their relations to comparative neurology, and in the July, 1881, Number of the *American Naturalist*, "On the Origin and Descent of the Human Brain," pointed out some hitherto neglected mechanical factors in the development of the organ of the mind and its osseous envelope.

While engaged in anatomical studies, the idea that there was a definite reason for everything, and that we might some day discover the reasons for many things not now known, was ever present to my mind. I could get half lights and glimpses of causes from hints in Henle, Holden, or Sharpey and Quain, and fancied I saw matters clearly enough in some particulars, only to be confused by contradictory experiences subsequently.

There seemed to be a definite enough law in the formation of valves in the veins, for instance, but every student was compelled to learn the location of these valves by arbitrary exercise of the memory. I think every student will conclude at the end of this paper that it is easy enough *now* to remember which veins are valved, and which are not. Let me present the subject just as it perplexed me at first. Nothing could be simpler, from the teleological standpoint, than that we should have valves in the veins of the arms and legs to assist the return of blood to the heart against gravitation, but what earthly use has a man for valves in the intercostal veins, which carry blood almost horizontally backward to the azygos veins? When recumbent, these valves are an actual detriment to the free flow of blood. The inferior thyroid veins, which drop their blood into the innominate, are obstructed by valves at their junction. Two pairs of valves are situated in the external jugular, and another pair in the internal jugular, but in recognition of their uselessness they do not prevent regurgitation of blood nor liquids from passing upwards.

An apparent anomaly exists in the absence of valves from parts where they are most needed, such as in the venæ cavæ, spinal, iliac, hæmorrhoidal, and portal. The azygos veins have imperfect valves.

Place man upon "all fours," and the law governing the presence and absence of valves is at once apparent, applicable, so far as I have been able to ascertain, to all quadrupedal and quadrumanous animals: *Dorsal veins are valved; cephalic, ventral, and caudal veins have no valves.* The apparent exceptions to this rule, I think, can be disposed of by considering the jugular valves as obsolescing, rendered rudimentary in man by the erect head, which in the lemur stage depended. The rudimentary azygos valves may be a recent creation, and an explanation of their presence may be found in the mutability of the cardinal system. The single Eustachian valve, being large in the fœtus, has a phylogenetic value. In this connection I would call attention to my mention, in *Science* (New York), June 25, 1881, of the probable brachial origin of the thyroid and thymus glands. There are many reasons for believing these bodies to be ruidmentary gills.

The only reason I can assign for the absence of cephalic and cervical valves generally, while the jugulars possess them, is, that the jugular system was the most important to our quadrupedal ancestors with dependent heads, hence valves developed in them, and that owing to the cranial blood vessels developing, *pari passu*, with the cranium and its contents generally, largely after man had assumed the erect position, the valvular formation elsewhere in the head would not occur while the jugular valves became rudimentary.

Certainly valves in the hæmorrhoidal veins would be out of place in quadrupeds, but to their absence in man many a life has been and will be sacrificed, to say nothing of the discomfort and distress occasioned by the engorgement known as piles, which the presence of valves in these veins would obviate. The spermatic valves are as useful in man as in other animals.

A glance at the accompanying diagram will afford an idea of the confusing distribution of valved and unvalved veins in the human being.

*a*, refers to the spinal system; *b*, jugular and caval to femoral; *c*, brachial; *d*, intercostal.

The position assumed by these valved veins when man is placed on all fours, corresponds with those to be found in quadrupeds, thus :

A noticeable departure from the rule obtaining in the vascular system of mammalia also occurs in the exposed situation of the femoral artery in man. The arteries lie deeper than the veins, or are otherwise protected for the purpose, the teleologist would say, of preventing hæmorrhage by superficial cuts. From the evolutionary standpoint it would appear that only animals with deeply-placed arteries would survive and transmit their peculiarities to their offspring, as the ordinary abrasions to which all animals are subject, not to mention their fierce onslaughts upon one another, would quickly kill off animals with superficially located arteries. But when man assumed the upright posture, the femoral artery, which was placed out of reach on the inner part of the thigh, became exposed, and were it not that this defect is nearly fully atoned for by his ability to protect the exposed artery in ways the brute could not, he too would have become extinct. Even as it is, this aberration is a fruitful cause of trouble and death.

Another disadvantage which occurs in the upright position of man is his greater liability to inguinal hernia. Quadrupeds have the main weight of abdominal viscera supported by ribs and strong pectoral and abdominal muscles. The weakest part of the latter group of muscles is in the region of Poupart's ligament, above the groin. Inguinal hernia is rare in other vertebrates because this weak part is relieved of the visceral stress, but as the pelvis receives the intestinal load in man, an immense number of tissues are manufactured to supplement this deficiency. It has been estimated that 20 per cent. of the human family suffer in this way, and strangulated hernia frequently occasions death.

If man has always been erect from creation, then we have nothing to hope from the future by way of an alteration of this defect. The same percentage of humanity will suffer to the end of time; but considered mechanically, the so-called conservative influence of nature, which will tend to pile up additional muscular tissue in this region by reason of the increased blood supply to that part, aided by natural and sexual selection, will eventually reduce the percentage of ruptures greatly, if it does not eventually correct the trouble altogether. The liability to femoral hernia is similarly increased by the upright position.

The peritoneal ligaments of the uterus subserve suspensorial functions in quadrupeds fully, which require much ingenious speculation to be faintly seen in man. The anterior, posterior and lateral ligaments are mainly concerned in preventing the gravid uterus from pitching too far toward the diaphragm of four-footed animals. The round ligaments are absolutely meaningless in the human female, but in lower animals serve the same purpose as the other ligaments. Prolapsus uteri, by the erect position and absence of support fitted to that attitude, are thus rendered frequent, to the destruction of health and happiness of multitudes.

As a deduction from mechanical laws, it could easily be imagined that an animal or race of men which had the longest maintained the erect position would have straighter abdomens, widely flared pelvic brims with contracted pelvic outlets, and that the weight of the spinal column would carry the sacrum lower down, and in general terms we find this to be the case. In quadrupeds the box-shaped pelvis, which admits of easy parturition, prevails, but where the position of the animal is such as to throw the weight of the viscera into the pelvis, the brim necessarily widens, these weighty organs sink lower, and the heads of the femora, acting as fulcra, admit of the crest of the ilium being carried outward, while the lower part of the pelvis must be contracted. This box shape exists in the child's innominate bones, while its protruding abdomen resembles that of the gorilla. The gibbon exhibits this iliac expansion through the sitting posture, which developed his ischial callosities. Similarly, iliac expansion occurs in the chimpanzee. The megatherium had wide iliacal expansion, due to its semi-erect habits, but as its weight was mainly supported by the huge tail with femora resting in acetabula placed far forwards, the leverage necessary to contract the lower pelvis is absent. Professor Weber, of Bonn, noted by Carl Vogt, "Vorlesungen über den Menschen," etc., distinguished four chief forms of the pelvis in man ; the oval, round, square and cuneiform, owned in order by Europeans, native Americans, Mongols and black races. Resting upon its own merits as an osseous mechanical proposition, it would seem that the older the race the lower the sacrum, and the greater the tendency to approximate the larger transverse diameter of the European female. The antero-posterior diameter of the simian pelvis is usually greater than the transverse ; a similar condition affords the cuneiform, from which could be inferred that the erect position in the negro races had not been so long maintained as by the Mongols, whose pelvis assumed the quadrilateral shape owing to persistence of spinal axis weight through greater time; this pressure has finally culminated in pressing the sacrum of the European nearer the pubes, with consequent lateral expansion at the expense of the antero-posterior or conjugate. From marsupialia to lemuridæ the box shaped pelvis persists, but with the wedge shape induced in man a remarkable phenomenon also occurs in the increased size of the fœtal head, in disproportion to the contraction of the pelvic outlet. While the marsupial head is about one-sixth the size of the smallest part of the parturient bony canal, the moment we pass to erect animals the greater relative increase is there in the cranial size, with coexisting decrease in the area of the outlet. This altered condition of things has caused the death of millions of otherwise perfectly healthy and well-formed human mothers and children. The palæontologist might tell us whether some such phenomenon of ischial approximation by natural mechanical causes has not caused the probable extinction of whole genera of vertebrates. If we are to believe that for our original sin the pangs of labor at term were increased, and also to believe in the disproportionate contraction of the pelvic space being an efficient cause of the same difficulties of parturition, the logical inference is inevitable that man's original sin consisted in his getting upon his hind legs.

Something of the changes noticed in the angle at which the head of the femur is set upon the shaft at different ages, is also noticeable phylogenetically. The neck of the femur in the child is obliquely placed, but in the adult is less so, and in advanced age tends to form a right angle with the socket. Both in the advance of age in the individual and the tendency of an animal to assume more and more the upright posture, this change of angle seems attributable to no other cause than bodily weight against the femoral heads.

This subject is not without direct application. Gynæcologists cause their patients to assume what is called the knee chest position, a prone one, for the purpose of restoring uteri to something near a natural position. Brown-Sequard recommends drawing away the blood from the spine in myelitis, or spinal congestion, by placing the patient on his abdomen or side with hands and feet somewhat dependent. The liability to spina-bifida is greatest in the human infant through the stress thrown upon the spine, and the absence of delivery troubles among lower races has reference to discrepancy between pelvic and cranial sizes not having been reached by those races. The Sandwich island mother has difficult delivery only when her progeny is half white, that breed being larger in the forehead than the native child.

The mechanism of the body, when fully recognized as mechanism and nothing else, and as governed by mechanical laws, physical as well as chemical influences, will place forthcoming physiological studies upon a broader, safer foundation, and result in grand generalizations. The hydro-dynamics of animal life would alone furnish a theme for thousands of investigators. At present the world goes on in its blindness, apparently satisfied that everything is all right because it exists at all, ignorant of the evil consequences of apparently beneficent peculiarities, vaunting man's erectness and its advantages, while ignoring the disadvantages. The observation that the lower the animal the more prolific, would eventuate the belief that the higher the animal the more difficulties encompass his development and propagation, and the cranio-pelvic incompatibility alone may settle the Malthusian doctrine effectually for the higher races of men through their extinction.—*American Naturalist.*

---

M. DURVIX, a French medical officer of the army, says that the sole cause of the diminishing population in France lies in the abuse of tobacco. But who knows?

DR. CHARLES WILLIAM SIEMENS, the well-known scientist, engineer, and electrician, died in London, of rupture of the heart, November 20, aged 63.

J. MARION SIMS is said to have left ready for publication a story of Revolutionary times, entitled "Lydia Mackay and Col. Tarleton."

PROF. CHARCOT has been elected member of the French Academy of Sciences, in place of the late M. Jules Cloquet.

## Figures and Tables

**Figure f1:**
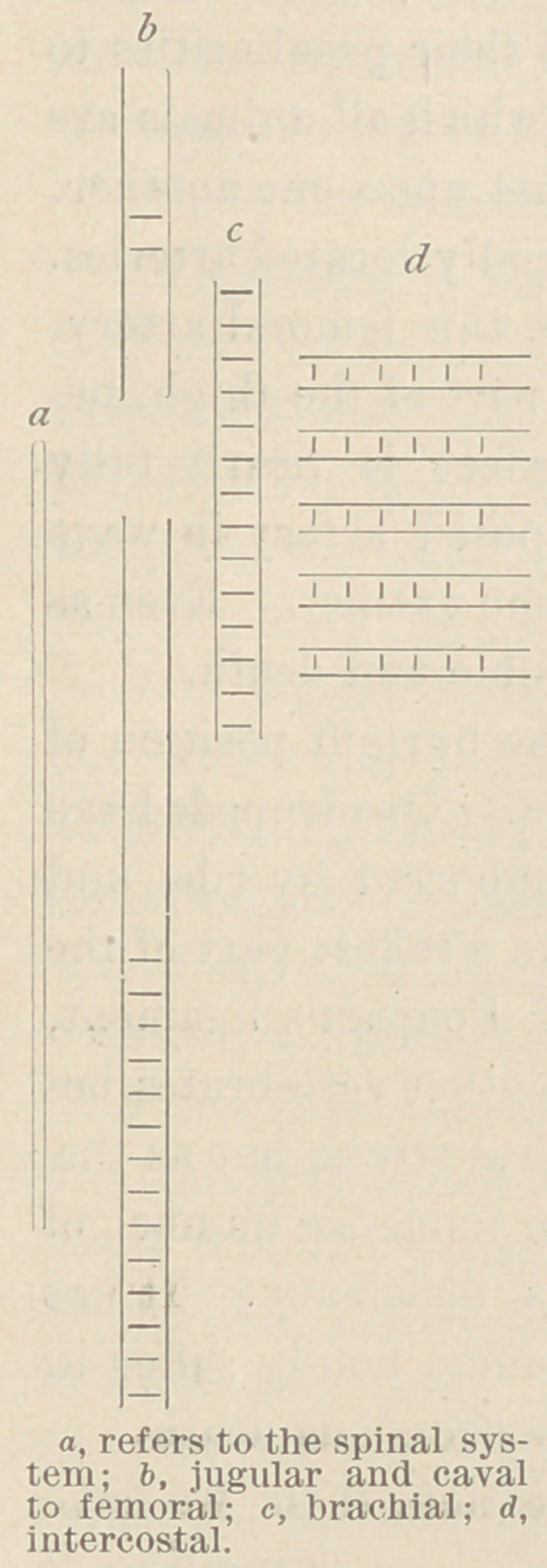


**Figure f2:**